# Small Molecule, Non-Peptide p75^NTR^ Ligands Inhibit Aβ-Induced Neurodegeneration and Synaptic Impairment

**DOI:** 10.1371/journal.pone.0003604

**Published:** 2008-11-03

**Authors:** Tao Yang, Juliet K. Knowles, Qun Lu, Hong Zhang, Ottavio Arancio, Laura A. Moore, Timothy Chang, Qian Wang, Katrin Andreasson, Jayakumar Rajadas, Gerald G. Fuller, Youmei Xie, Stephen M. Massa, Frank M. Longo

**Affiliations:** 1 Department of Neurology and Neurological Science, Stanford University, Stanford, California, United States of America; 2 Department of Neurology, University of North Carolina-Chapel Hill, Chapel Hill, North Carolina, United States of America; 3 Department of Anatomy and Cell Biology, The Brody School of Medicine at East Carolina University, Greenville, North Carolina, United States of America; 4 Department of Pathology and Taub Institute, Columbia University, New York, New York, United States of America; 5 Department of Chemical Engineering, Stanford University, Stanford, California, United States of America; 6 Department of Neurology and Laboratory for Computational Neurochemistry and Drug Discovery, San Francisco Veterans Affairs Medical Center, and Department of Neurology, University of California San Francisco, San Francisco, California, United States of America; University of Cambridge, United Kingdom

## Abstract

The p75 neurotrophin receptor (p75^NTR^) is expressed by neurons particularly vulnerable in Alzheimer's disease (AD). We tested the hypothesis that non-peptide, small molecule p75^NTR^ ligands found to promote survival signaling might prevent Aβ-induced degeneration and synaptic dysfunction. These ligands inhibited Aβ-induced neuritic dystrophy, death of cultured neurons and Aβ-induced death of pyramidal neurons in hippocampal slice cultures. Moreover, ligands inhibited Aβ-induced activation of molecules involved in AD pathology including calpain/cdk5, GSK3β and c-Jun, and tau phosphorylation, and prevented Aβ-induced inactivation of AKT and CREB. Finally, a p75^NTR^ ligand blocked Aβ-induced hippocampal LTP impairment. These studies support an extensive intersection between p75^NTR^ signaling and Aβ pathogenic mechanisms, and introduce a class of specific small molecule ligands with the unique ability to block multiple fundamental AD-related signaling pathways, reverse synaptic impairment and inhibit Aβ-induced neuronal dystrophy and death.

## Introduction

Slowing the progression of Alzheimer's disease (AD) will likely require parallel strategies of managing amyloid-beta (Aβ) levels and reducing neuronal vulnerability to Aβ. With regard to neuroprotective strategies, the p75 neurotrophin receptor (p75^NTR^) is an attractive target [1], [2]. Binding of neurotrophins, including nerve growth factor (NGF), to p75^NTR^ promotes pro-apoptotic or pro-survival signaling, depending on the recruitment of survival- versus death-promoting adaptors [3], [4]. p75^NTR^ is expressed in adult brain primarily by basal forebrain cholinergic neurons, but also by hippocampal, entorhinal and neocortical neurons, each vulnerable in AD (reviewed in [5]). Moreover, p75^NTR^ expression is upregulated in cortical [6] and hippocampal [7] tissue in AD. Increased p75^NTR^ and decreased Trk neurotrophin receptor levels in AD, along with studies showing that increased p75^NTR^/Trk ratios lead to neuronal degeneration, further encourage therapeutic targeting of p75^NTR^ [4].

Substantial overlaps exist between p75^NTR^-mediated signaling and degenerative signaling in AD. In AD brain and in cultured neurons treated with Aβ, there is excessive activation of calpain/cdk5 [8], GSK-3β [9], and JNK and its downstream transcriptional activator c-Jun [10]. p75^NTR^ stimulates calpain activation through its Chopper cell death domain, [11] and mediates NGF-induced inhibition of GSK-3β [12]; in addition, it can mediate induction of cell death through the activation of JNK [13]. Further, it has been reported that Aβ can bind to p75^NTR^, and the receptor mediates Aβ-induced cell death, in part by induction of c-Jun activation [1], [14], [15]. Thus, there are numerous potential points of direct and indirect interactions between p75^NTR^ and AD pathogenic mechanisms.

p75^NTR^ represents a significant target for AD therapeutic development from a number of perspectives. To the extent that direct interactions between p75^NTR^ and Aβ contribute to AD, preventing those interactions could inhibit neurodegeneration. Further, modulating the receptor to reduce activation of c-Jun and calpain activity could counteract Aβ activation of these signaling intermediates. Moreover, since Aβ down-regulates trophic signaling, particularly the PI3K/AKT pathway which promotes survival and is important for synaptic function [16], the promotion of AKT activation by ligand binding to p75^NTR^ [3], [17] may reduce the effects of Aβ In addition, AKT down-regulates JNK [18] and GSK3β [19]. Thus, targeting p75^NTR^ could protect neurons from Aβ by at least three possible mechanisms: i) blocking a deleterious interaction between Aβ and p75^NTR^; ii) down-regulating deleterious signaling (calpain, GSK3β and c-Jun) which mediates Aβ toxicity; iii) upregulating survival signaling (AKT) which is normally inhibited by Aβ and which can antagonize Aβ mechanisms. The latter two mechanisms could be operative even under circumstances in which Aβ causes degeneration independent of p75^NTR^.

Aberrant activation of the cdk5, GSK3β and JNK kinases leads to tau hyperphosphorylation, cytoskeletal disruption and neuritic dystrophy [20]. Also, excessive activation of these kinases along with Aβ-induced inhibition of CREB activation causes synaptic dysfunction [21]–[23]. Thus, small molecules inhibiting activation of cdk5, GSK3β, JNK and/or c-Jun have become important therapeutic candidates [20], [24], [25]. However, it seems unlikely that modulating a single target will provide an effective therapy; in addition, given the ubiquity of these targets and their functions in a very broad range of cell types it may be anticipated that adverse effects will limit their utility. Therefore, the possibility of inhibiting Aβ-induced excessive activation of cdk5, GSK3β and c-Jun through a receptor expressed by neurons particularly vulnerable in AD is an attractive strategy for therapeutic development.

Expanding on development of synthetic peptides modeled on loop 1 of NGF that prevent neuronal death through p75^NTR^-mediated mechanisms [26], we identified small molecule, non-peptide ligands, with favorable pharmaceutical properties, that bind specifically to p75^NTR^ and activate survival-promoting signaling, including AKT, in hippocampal neurons ([17], reviewed in [5]). In the present study, we tested the hypothesis that these ligands would interfere with deleterious Aβ signalling and its functional consequences. We demonstrate that these compounds inhibit Aβ-induced neuronal death, neuritic degeneration and activation of calpain/cdk5, GSK3β, and c-Jun; and reverse Aβ-mediated inhibition of AKT and CREB activation, and synaptic function. These findings suggest that the use of small molecule p75^NTR^ ligands may be a therapeutically feasible approach to AD capable of simultaneously targeting multiple underlying pathogenic mechanisms.

## Results

### p75^NTR^ small molecule ligands inhibit Aβ-induced death of hippocampal, cortical and septal neurons

**Figure 1:**
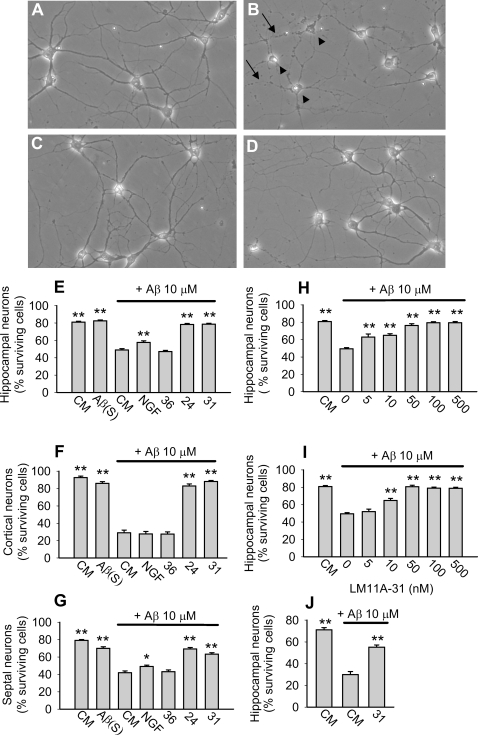
p75^NTR^ small molecule ligands protect neurons from Aβ-induced death. 6–7 DIV hippocampal, cortical and septal neurons were exposed to: culture medium alone (CM); CM containing 10 µM Aβ(S), a control peptide containing Aβ(1-42) residues in a scrambled sequence; 10 µM Aβ alone or with NGF (100 ng/ml , ∼4 nM), LM11A-24, -31, or -36 (100 nM). After 72 hours, cultures were fixed and photographed under phase contrast microscopy. (A) In CM alone, neurons had round, phase bright cell bodies with outgrowth of intact neurites (normal morphology). (B) In the presence of Aβ, many neurons exhibited the degenerative findings of cell body shrinkage (arrowheads), vacuolated cytoplasm, and neurite beading and fragmentation (arrows). (C, D) Neurons co-treated with Aβ and LM11A-24 (C) or LM11A-31 (D) exhibited normal morphology. (E–J) Neuronal survival was quantitated using morphological criteria (see Methods) and survival in each experimental condition was statistically compared to Aβ alone. In cultures of hippocampal (E) cortical (F) and septal (G) neurons, Aβ significantly decreased survival while Aβ (S) had no effect. LM11A-24 and -31 inhibited Aβ-induced death while NGF and LM11A-36 negative control compound demonstrated no protective effects with the exception of NGF exhibiting a small protective effect in hippocampal and septal cultures (for hippocampal cultures, n = 45–146 fields counted over 5–16 separate experiments; for cortical cultures, n = 25–40 fields counted over 5 separate experiments; for septal cultures, n = 50–60 fields counted over 6 separate experiments). (H, I) 6–7 DIV hippocampal neurons were exposed to CM alone or Aβ in the presence or absence of LM11A-24 (H) or -31 (I) at the indicated concentrations. LM11A-24 and -31 were protective against Aβ in a dose-dependent manner, with an EC_50_ of approximately 20 nM (LM11A-24: n = 20–146 fields counted over 3–16 separate experiments. LM11A-31: n = 43–146 fields counted over 5–16 separate experiments). (J) 6–7 DIV hippocampal neurons were exposed to CM alone or 10 µM fibrillar Aβ±LM11A-31. LM11A-31 inhibited fibril-induced neuronal death (n = 30 fields counted over 3 separate experiments).

Ligands LM11A-24 and LM11A-31 were selected from p75^NTR^ ligands developed by our laboratories based on chemical and pharmacological features favorable for drug development [17]. LM11A-31 is an isoleucine derivative (MW 243.3) and LM11A-24 is a caffeine derivative (MW 322.4). LM11A-36 is structurally similar to LM11A-24 but is inactive in neurotrophic assays [17] and served as a negative control (**Fig. S1**). 6–7 DIV hippocampal, cortical and septal neurons were treated with oligomeric Aβ in the presence or absence of p75^NTR^ small molecule ligands or NGF. Preliminary experiments demonstrated that oligomeric Aβ (derived from each of three oligomeric Aβ protocols as described in **Methods** and **Fig. S2**) reached maximum toxicity (neuronal death) at 5–10 µM (data not shown), a concentration range similar to that reported by other laboratories. Exposure of neurons to Aβ resulted in somal shrinkage and vacuolation along with neurite fragmentation and beading; whereas the majority of neurons co-treated with Aβ and 100 nM LM11A-24 or LM11A-31 exhibited a normal appearance with intact cell bodies and neurites (**Fig. 1A–D**). Quantitative analysis of hippocampal, cortical, and septal neuronal cultures demonstrated that Aβ induced a 40–65% reduction in survival while simultaneous addition of LM11A-24 or LM11A-31 prevented 70–90% of the Aβ-induced decrease in survival. NGF and LM11A-36 had no protective effect except for a minor protective effect by NGF in hippocampal and septal cultures **(** **Fig. 1E, G** **)**. In dose-response studies, LM11A-24 and -31 inhibited Aβ-induced death with EC_50_ values of ∼20 nM, with the protective effect persisting to at least 500 nM (**Fig. 1H, I**). LM11A-31 was also found to be protective against Aβ fibrillar preparations (**Fig. 1J**).

**Figure 2:**
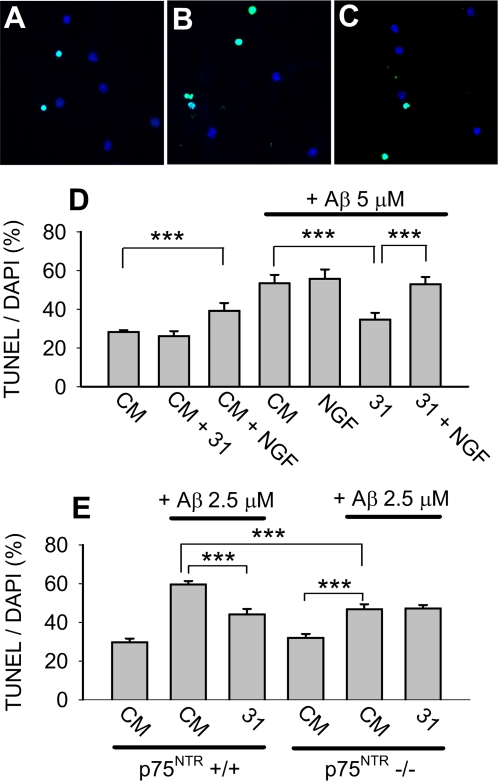
LM11A-31 inhibition of Aβ-induced toxicity is p75^NTR^ dependent. (A–C) 6–7 DIV hippocampal neurons were treated with: (A) CM alone; (B) 5 µM Aβ; (C) 5 µM Aβ+100 nM LM11A-31 for 72 hours and then stained with TUNEL/DAPI. Many neurons exposed to Aβ were TUNEL-positive (green), indicative of death; whereas the majority of neurons co-treated with Aβ and LM11A-31 were TUNEL-negative. (D) Quantitation of the percentage of TUNEL-positive neurons demonstrated that LM11A-31 alone had no effect on baseline death while NGF (100 ng/ml) was associated with a significant increase in death. Aβ caused an approximate 2.0-fold increase in death that was significantly inhibited by LM11A-31 but not NGF. In the presence of NGF, LM11A-31 failed to prevent Aβ-induced death (n = 98–131 fields derived from a total of 6 separate experiments). (E) In 6–7 DIV hippocampal neuronal cultures derived from C57Bl/6 p75^NTR^+/+ and −/− mice, Aβ triggered a 2.0-fold increase in death in p75^NTR^+/+ cultures that was significantly inhibited by LM11A-31. In p75^NTR^−/− cultures, Aβ triggered a significant 1.5-fold increase in death, a degree of increase significantly less than that found in +/+ cultures. In p75^NTR^−/− cultures, LM11A-31 demonstrated no protective effect (n = 69–140 fields per condition, derived from a total of 4 separate p75^NTR^−/− cultures and 6 separate p75^NTR^+/+ cultures).

To confirm that p75^NTR^ ligands prevent Aβ-induced neuronal death, hippocampal neuronal survival was further assessed using Terminal deoxynucleotidyl Transferase Biotin-dUTP Nick End Labeling (TUNEL)/DAPI staining. Neurons treated with Aβ exhibited increased TUNEL staining whereas fewer neurons co-treated with Aβ and LM11A-31 demonstrated TUNEL signal (**Fig. 2A–C**). Quantitative analyses (**Fig. 2D**) demonstrated that in the absence of Aβ, addition of LM11A-31 had no significant effect on neuronal survival while NGF was associated with a small but statistically significant increase in death. These findings were consistent with previous studies showing that under certain culture conditions, NGF promotes death of hippocampal neurons [27]. LM11A-31 inhibited Aβ-induced death while NGF had no protective effect. The ability of LM11A-31 and LM11A-24 to block Aβ-induced death was further confirmed using Hoechst staining (**Fig. S3**).

### Small molecule ligand protection is mediated through p75^NTR^

Previous studies [17] demonstrated specificity of LM11A-24 and -31 for p75^NTR^. The ability of p75^NTR^ ligands to induce PI3K/AKT activation was entirely dependent on p75^NTR^ and these ligands did not activate Trk receptors [17]. These previous studies also demonstrated differential activities of neurotrophins and the small molecule ligands on p75^NTR^-expressing cells. In cultures of oligodendrocytes, which express p75^NTR^ but not Trk receptors, NGF or proNGF can induce cell death; however, LM11A-24 and -31 not only failed to promote death but blocked neurotrophin-induced death. In the current study, we reasoned that if a compound promotes its protective effect through a ligand-type interaction with p75^NTR^, this effect should be blocked by the addition of a receptor-saturating concentration of a competing, non-protective ligand, such as NGF. The addition of NGF (100 ng/ml) together with LM11A-31 (100 nM) reversed entirely the protective effect of LM11A-31 (**Fig. 2D**), consistent with a model in which these compounds act through a common receptor. An alternative explanation is that toxicity induced by NGF might mask a non- p75^NTR^-dependent protective mechanism of LM11A-31. As a second approach for determining whether the LM11A-31 protective effect was mediated through p75^NTR^, assays were conducted using p75^NTR^+/+ and p75^NTR^−/− hippocampal neurons derived from mice maintained on a C57Bl/6-strain background. Using neurons from that strain, maximum Aβ-induced death was reached at 2.5 µM Aβ and this dose was therefore used for subsequent studies. In p75^NTR^+/+ cultures, Aβ triggered a 2-fold increase in death that was significantly inhibited by LM11A-31, a protective effect consistent with earlier studies (**Fig. 2E**). In p75^NTR^−/− cultures, significant Aβ toxicity was reduced to a 1.5-fold increase in death. The significant difference in the degree of cell death triggered in p75^NTR^ wildtype and mutant cultures indicated that the presence of wildtype p75^NTR^ contributes to Aβ-induced toxicity. Notably, in p75^NTR−/−^ cultures LM11A-31 had no protective effect, indicating a protective mechanism requiring p75^NTR^.

### p75^NTR^ small molecule ligands prevent Aβ-induced neuronal death in organotypic hippocampal slice cultures

**Figure 3:**
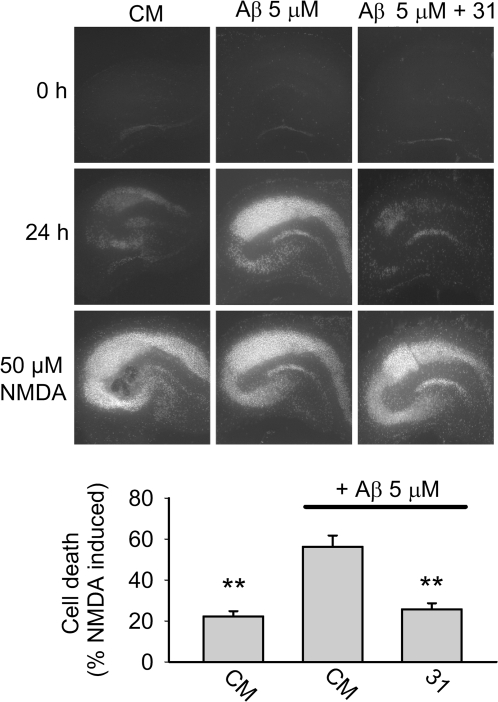
LM11A-31 inhibits Aβ-induced death of hippocampal neurons in postnatal organotypic slice cultures. Organotypic slice cultures were prepared from PND-8 rat brain and allowed to mature *in vitro* for 11–19 days. Pyramidal neuron death was detected by propidium iodide (PI) staining, and all experimental conditions were compared to Aβ treatment alone. Upper row, at baseline only trace levels of PI staining were detected. Middle row, PI staining after a 24 hour exposure to either culture medium (CM), Aβ or Aβ+LM11A-31 at 100 nM demonstrates readily apparent Aβ-induced pyramidal neuron death that is inhibited in the presence of p75^NTR^ ligand. Bottom row, PI staining shows maximum neuronal death after 24 hour treatment with NMDA. In the lower panel, quantitative analysis of PI staining demonstrates a significant reduction in Aβ-induced neuronal death (n = 56–59 brain slices derived from 4 independent studies).

Organotypic slice culture derived from postnatal brain and matured *in vitro*, is a widely used model for the study of neurodegenerative mechanisms and potential therapeutics. Addition of Aβ to rat postnatal hippocampal slice cultures leads to death of pyramidal neurons as detected by propidium iodide (PI) uptake [28]. In our studies, 24 hour Aβ exposure led to markedly increased in PI staining by pyramidal neurons which was blocked entirely by co-treatment with LM11A-31 (**Fig. 3**).

### p75^NTR^ small molecule ligands prevent Aβ-induced neuritic dystrophy in matured neurons

**Figure 4:**
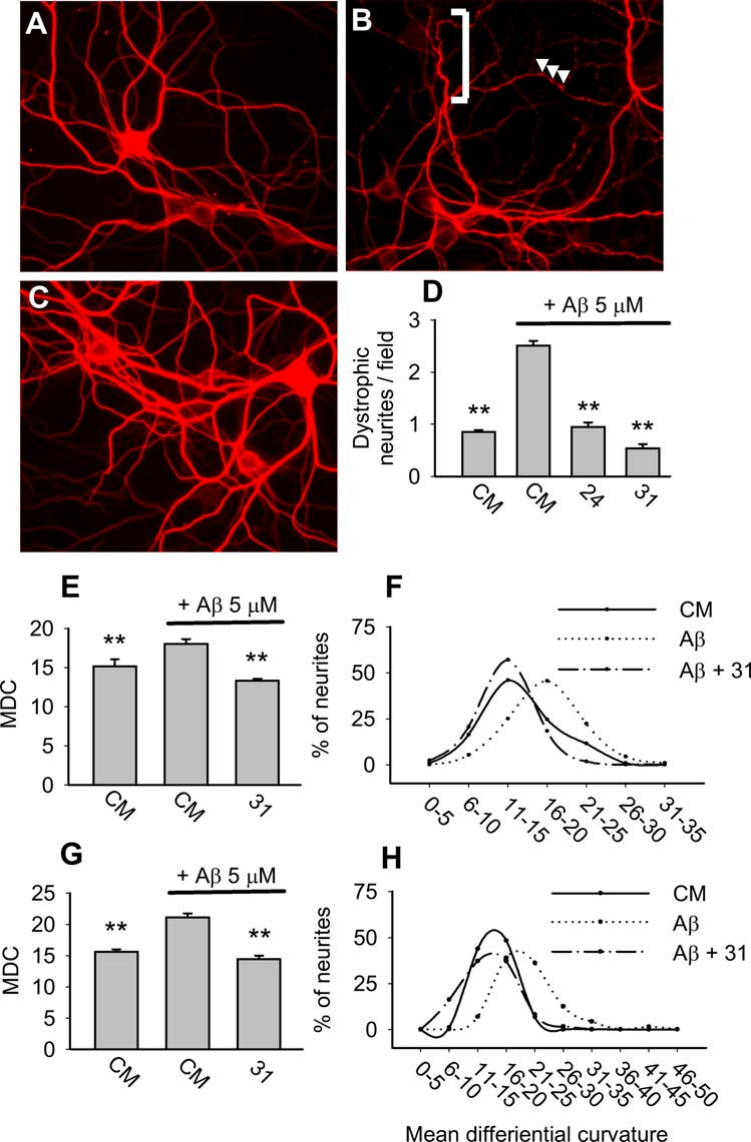
p75^NTR^ small molecule ligands prevent Aβ-induced neuritic dystrophy. 21–22 DIV hippocampal neurons were exposed to the following conditions: (A) culture medium (CM); (B) 5 µM oligomeric-NaOH-derived Aβ; or (C) 5 µM oligomeric-NaOH-derived Aβ+100 nM LM11A-31. After 48 hours, cultures were fixed and immunostained for MAP2 to visualize dendrites. In the presence of CM alone, dystrophic changes including beading and tortuosity were rare. In contrast, Aβ induced beading (arrowheads) and increased tortuosity (brackets), and each of these changes was markedly reduced with co-administration of LM11A-31. (D) Dystrophic neurites, defined as neurites exhibiting beading and/or multiple abrupt turns (i.e., tortuosity) were measured by a blinded observer. Data is expressed as average numbers of dystrophic neurites per field. All conditions were compared to Aβ alone (n = 8 randomly chosen fields from 3 independent experiments). (E) Mean differential curvature (MDC) analysis in randomly selected fields demonstrated that oligomeric-NaOH-derived Aβ induced a significant increase in MDC which was prevented by LM11A-31 (n = 7–9 fields per condition). (F) Distribution analysis of MDC values (X axis) demonstrates a rightward shift in the presence of oligomeric-NaOH-derived Aβ that was mitigated in the presence of LM11A-31. (G–H) The same assays and analyses were performed using 5 µM oligomeric-HFIP-derived Aβ which demonstrated similar findings (n = 9 fields per condition derived from 3 independent experiments).

Aβ-induced tau/cytoskeletal derangement causes neuritic dystrophy, a process which occurs in early stages of AD [29] and is characterized by the appearance of varicosities and excessive tortuosity [30], [31]. Hippocampal neurons kept *in vitro* for ≥3 weeks express mature isoforms of tau protein, and when exposed to Aβ primarily demonstrate neuritic dystrophy rather than death [30]. Treatment of DIV 21–22 hippocampal neurons with 5 µM Aβ induced dystrophic changes which were prevented almost entirely by LM11A-24 and -31 (**Fig. 4A**–**D**). Assessment of dystrophy by visual criteria (**Fig. 4D** **)** and by quantitation of the neurite mean differential curvature, a measure of tortuosity (**Fig. 4E**–**H**), showed that these ligands effectively blocked Aβ-induced dystrophy. This finding suggests that the compounds may interfere at an early/upstream stage with the complex cascade of Aβ-induced signaling [20] which results in the disruption of neuritic integrity.

### p75^NTR^ small molecule ligands prevent Aβ-induced AKT inactivation and their protective effect is dependent, in part, on PI3K activity

**Figure 5:**
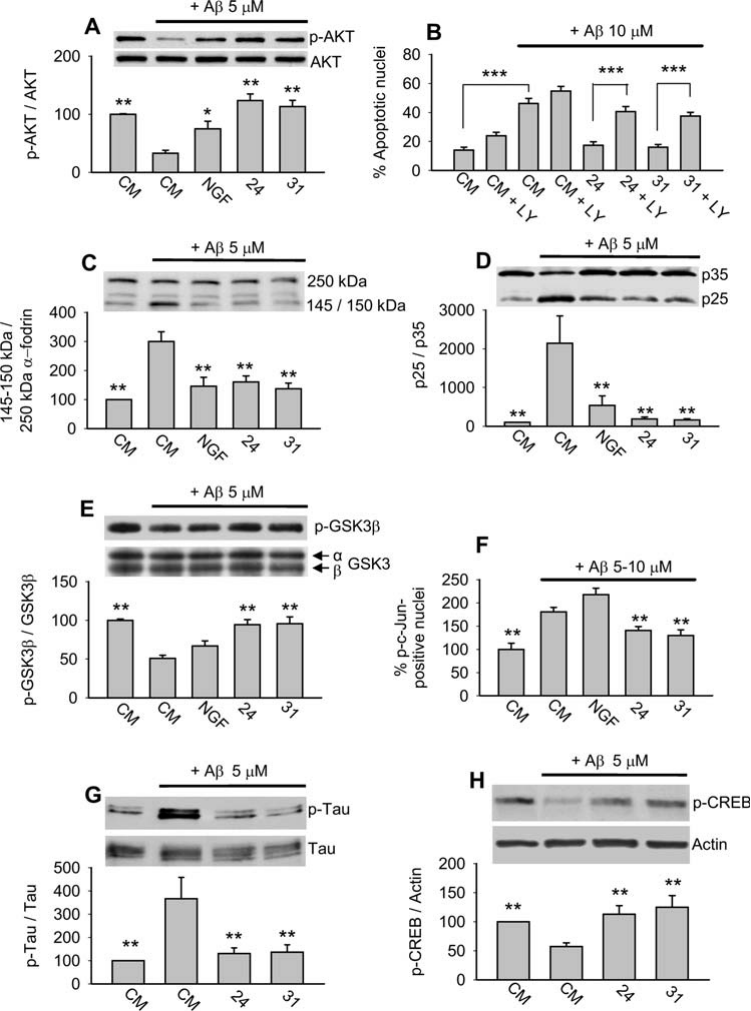
Modulation of Aβ-induced deleterious signaling. For each study, LM11A-24 and -31 were present at 100 nM and NGF at 50 or 100 ng/ml. All conditions were compared to Aβ treatment alone, unless indicated otherwise (e.g. Fig. 5B). For the majority of protein preparations, each derived from separate experiments; two independent Western analyses were conducted. (A) DIV 21–22 hippocampal neurons were treated with CM alone, CM+Aβ, or Aβ in the presence of LM11A-24, -31 or NGF and examined at the 4 hour time point. AKT activation was significantly decreased by Aβ, while LM11A-24, -31 and NGF prevented this decrease. The ability of NGF to prevent Aβ-induced AKT inhibition was diminished relative to that of the small molecules (n = 8–14 separate Western blots from 9 independent protein preparations). (B) Neurons were treated with CM alone or with the indicated combinations of the PI3K inhibitor LY294002, Aβ and LM11A-24 or -31. After 72 hours, cultures were stained with Hoechst 33258 for cell death quantitation. In CM, LY294002 increased cell death by approximately 1.7-fold without reaching significance. In the presence of Aβ, LY294002 blocked the protective effect of p75^NTR^ ligands and resulted in a > 2.0-fold increase in cell death (n = 35–45 fields derived from a total of 4–5 experiments for each condition). (C–E) DIV 21–22 hippocampal neurons were treated with CM or Aβ in the presence of LM11A-24, -31 or NGF for 4 hours. (C) Addition of Aβ resulted in increased calpain-induced cleavage of full-length α-fodrin (250 kDa) to its 150 and 145 kDa fragments. Co-treatment with LM11A-24, -31 or NGF, significantly reduced Aβ-induced calpain activation (n = 4–7 separate Western blots from 4 independent protein preparations). (D) Addition of Aβ induced cdk5 activity as revealed by the increased ratio of the cleaved (p25) to uncleaved (p35) regulatory subunit. Activation was inhibited by LM11A-24, -31 or NGF (n = 8–10 separate Western blots from 7 independent protein preparations). (E) Addition of Aβ induced GSK3β activation as revealed by a decrease in the ratio of p-GSK3β^Ser9^ signal to total GSK3β. Activation was significantly inhibited by LM11A-24 and 31, but not NGF (n = 8–14 separate Western blots from 7 independent protein preparations). (F) In DIV 6–7 neuronal cultures examined at the 12 hour time point, Aβ induced a 1.8-fold increase in the proportion of phospho-c-Jun positive nuclei, a measure of JNK/c-Jun activation. Activation was significantly inhibited by LM11A-24 and 31, but not NGF (n = 139–210 fields from 6 individual experiments). (G) In DIV 21–22 hippocampal neuronal cultures examined at the 4 hour time point, Aβ induced a 3.5-fold increase in Tau^Ser202^ phosphorylation which was significantly inhibited by LM11A-24 and -31 (n = 10 separate Western blots from 5 independent protein preparations). (H) In DIV 21–22 hippocampal neuronal cultures examined at the 3 hour time point, Aβ significantly decreased CREB phosphorylation by 43%, while this decrease was prevented by LM11A-24 and -31 (n = 10 separate Western blots from 5 independent protein preparations).

In previous studies with hippocampal neurons, LM11A-24 and -31 were found to activate AKT in a p75^NTR^-dependent manner and their neurotrophic effect was dependent upon PI3K [17]. These findings raised the possibility that these ligands might counteract the ability of Aβ to decrease levels of AKT activation. Treatment of 21–22 DIV hippocampal neurons with Aβ induced a significant decrease in AKT activity that was blocked by LM11A-24, -31 and NGF, although the effect of NGF was less than that of the small molecules (**Fig. 5A**). To determine whether the small molecule protective effect was dependent upon PI3K/AKT activity, we co-treated 6–7 DIV hippocampal neurons with Aβ, LM11A-24 or -31, and LY294002, an inhibitor of PI3K. Under baseline conditions (**Fig. 5B**), LY294002 induced a 1.7-fold increase in cell death without reaching significance, a trend consistent with the known role of PI3K/AKT signaling in promoting neuronal survival [3]. In the presence of Aβ without LY294002, LM11A-24 and -31 blocked the Aβ-death promoting effect consistent with earlier results. However, in the presence of LY294002, the protective effect of LM11A-24 and -31 was significantly reduced. The ability of LY294002 to block the p75^NTR^ ligand protective effect, along with the finding that p75^NTR^ ligands reverse Aβ-induced inhibition of AKT activation, suggest that maintenance of PI3K/AKT signaling by LM11A-24 and -31 contributes to their protective effect.

### p75^NTR^ small molecule ligands inhibit Aβ-induced activation of calpain/cdk5, GSK3β and c-Jun

Activation of calpain results in the cleavage of the 250 kDa cytoskeletal protein α-fodrin to 145 and 150 kDa fragments, and the levels of these fragments serve as a measure of calpain activity [32]. Exposure of 21–22 DIV hippocampal neurons to Aβ triggered a nearly 3-fold increase in the ratio of fragmented to full-length α-fodrin forms (**Fig. 5C**). Of note, α-fodrin is also a substrate for caspase 3 in degenerative states, with cleavage to 150 kDa and 120 kDa forms [33]. Quantitation of 120 kDa α-fodrin fragments revealed no significant differences across conditions (data not shown) suggesting that Aβ-induced cleavage resulted from calpain rather than caspase activity. LM11A-24, -31 and NGF inhibited Aβ-induced α-fodrin cleavage, indicating that they prevented Aβ-induced calpain activation.

Activated calpain cleaves the cdk5 p35 regulatory subunit to the p25 constitutively active form promoting excessive cdk5 activation; the ratio of p25 to p35 reflects cdk5 activation [32]. Treatment of 21–22 DIV neurons with Aβ resulted in markedly increased p35 cleavage, as evidenced by a significant increase in the ratio of p25 to p35 (**Fig. 5D**). Co-treatment with LM11A-24, -31 or NGF prevented the increase in p25/p35 ratio, demonstrating that p75^NTR^ ligands inhibit Aβ-induced cdk5 activation.

Given that LM11A-24 and -31 promote PI3K/AKT signaling [17] and that this signaling inhibits GSK3β [19] and JNK activation [34], we determined whether these ligands, as well as NGF, prevent Aβ-induced GSK3β and c-Jun activation. GSK3β activity is increased when the Ser^9^ residue is dephosphorylated, providing a measure of the GSK3β activation state [19]. Addition of Aβ to 21–22 DIV cultures induced significant dephosphorylation (i.e., activation) of GSK3β, while co-administration with LM11A-24 and -31, but not NGF, prevented this activation (**Fig. 5E**). An established method for monitoring JNK and c-Jun activation consists of quantitating neuronal nuclei positive for phospho-c-Jun immunostaining [35]. Treatment of 6–7 DIV neurons with Aβ resulted in activation of c-Jun, which was largely inhibited by co-treatment with LM11A-24 or -31 but not by NGF (**Fig. 5F**).

In summary, LM11A-24 and -31, but not NGF, demonstrated significant inhibition of Aβ-induced GSK3β and c-Jun activation. In contrast, LM11A-24, -31 and NGF each inhibited the ability of Aβ to downregulate AKT signaling and to promote calpain/cdk5 signaling. These differences in signaling profiles between the small molecules and NGF might account, in part, for the greater protective effects of the small molecules and point to the importance of GSK3β and c-Jun activation in mediating Aβ-induced degeneration.

Since cdk5, GSK3β and JNK contribute to Aβ-induced tau phosphorylation [20], [24], [25], we determined whether LM11A-24 and -31 might also prevent tau phosphorylation. In 21–22 DIV neurons, exposure to Aβ resulted in a significant increase in tau phosphorylation at Ser^202^, a well characterized tau residue which is phosphorylated by each of these kinases and which is found to be phosphorylated in early AD [25], [36], [37]. Co-administration of LM11A-24 or -31 almost entirely prevented Aβ-induced tau Ser^202^ phosphorylation (**Fig. 5G**).

### p75^NTR^ small molecule ligands prevent Aβ-induced inactivation of CREB

In addition to aberrant activation of calpain/cdk5, GSK3β and JNK, another potential mechanism by which Aβ inhibits synaptic function is through inhibition of CREB, a fundamental contributor to long-term potentiation (LTP) [21]. To further examine the effects of LM11A-24 and -31 on Aβ-induced changes in signaling, we determined whether these ligands were capable of blocking Aβ-induced CREB deactivation. Treatment of 21–22 DIV hippocampal neurons with Aβ for 3 hours resulted in a 43% reduction in phosphorylation of CREB, consistent with previous studies [21]. Notably, co-treatment with 100 nM LM11A-24 or -31 blocked the inhibitory effect of Aβ on CREB phosphorylation (**Fig. 5H**).

### LM11A-31 corrects deficits in synaptic transmission in Aβ-treated hippocampal slices

**Figure 6:**
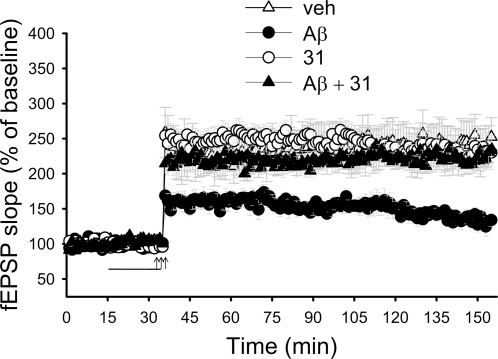
LM11A-31 rescues the deficit in CA1-LTP in Aβ-treated hippocampal slices. Extracellular field excitatory postsynaptic potential (fEPSP) was measured as described in Methods. Arrows indicate tetanus application. The horizontal bar indicates the period during which Aβ and/or LM11A-31 were added to the bath solution. Aβ did not affect baseline transmission. Application of LM11A-31 (100 nM) rescued Aβ-induced impairment of LTP without affecting baseline transmission. The values for the last data point in the graph are as follows: vehicle (veh, n = 8) 252.4±27.6%; Aβ (n = 7) 134.0±9.3%; LM11A-31 (n = 7) 230.2±24.3%.; Aβ+LM11A-31 (n = 7) 233.6±17.6%. Vehicle vs. Aβ, p = 0.02; Aβ vs. Aβ+LM11A-31, p = 0.0006; vehicle vs. LM11A-31, p = 0.8131.

The effect of the ligands on CREB phosphorylation strongly suggested that they might be able to reverse the Aβ-induced inhibition of LTP in the brain. Electrophysiological experiments were performed on hippocampal slices derived from adult mice that received a tetanus to produce LTP at the Shaffer collateral-CA1 connection. As previously shown [21], we found that the exposure of slices to Aβ (200 nM) for 20 min before tetanization led to significant impairment of LTP (**Fig. 6**). However, when slices were treated concomitantly with Aβ and LM11A-31 (100 nM), LTP was normalized. LM11A-31 alone did not affect potentiation.

## Discussion

There is no available therapy for AD that effectively targets underlying disease mechanisms. The present studies demonstrate that non-peptide small molecule ligands targeting p75^NTR^, a receptor upregulated in neurons vulnerable in AD, prevent Aβ-induced neuritic dystrophy and cell death, while inhibiting Aβ-induced tau phosphorylation and the activation of several key signaling intermediates, each a candidate therapeutic target in its own right. The inhibition of Aβ-induced and transgenic mouse-related synaptic impairment, the prevention of neuritic dystrophy and neuronal death, along with the modulation of multiple intracellular signaling mechanisms involved in AD is a novel activity profile for small molecule ligands acting at a known receptor target.

The protective effect of these compounds is likely mediated through p75^NTR^. Their effects on neuronal signaling and survival are p75^NTR^-dependent, they induce signaling-adaptor recruitment to p75^NTR^ and they fail to activate Trk receptors [17]. In addition, a standardized receptor binding screen conducted with LM11A-31 (Cerep panel, **Table S1**) failed to detect binding of p75^NTR^ ligands to other receptors. NGF lacked protective activity, and as expected for an ‘inactive’ ligand, inhibited the protective effect of p75^NTR^ small molecule ligands, consistent with their protective effect being mediated through p75^NTR^. Moreover, in assays employing p75^NTR−/−^ neurons in which Aβ-induced death was reduced but still present to a significant degree, small molecule ligand protective activity was entirely absent. Taken together, our prior and current studies indicate that these ligands inhibit Aβ-induced degeneration by interacting with p75^NTR^. The expression of p75^NTR^ by glial cells [38] raises the possibility that these ligands might also function through p75^NTR^-mediated effects on non-neurons; however, the relative lack of non-neuronal cells in the culture protocols applied here and the effects evident in short term signaling studies make this possibility unlikely.

There are several mechanisms by which p75^NTR^ ligands might inhibit Aβ toxicity. Aβ(1-40) aggregates bind to p75^NTR^ and induce cell death and a modified NGF loop 1 synthetic peptide was found to block NGF binding to p75^NTR^ and prevent Aβ(1-40)-induced death of cultured cortical neurons [14]. It has yet to be determined whether Aβ(1-42) oligomer species similarly bind to p75^NTR^ to induce neuronal degeneration. Our finding that Aβ(1-42) oligomer-induced death is significantly, though incompletely, decreased in p75^NTR^−/− cultures suggests that p75^NTR^ might be one of several targets mediating oligomeric Aβ(1-42) effects. Thus, small molecule p75^NTR^ ligands might prevent degeneration, in part, by preventing Aβ binding to p75^NTR^. Alternatively, p75^NTR^ might not be an important target for Aβ binding but might instead contribute to Aβ toxic effects through indirect mechanisms. Its ability to affect deleterious or survival-promoting signaling (i.e. c-Jun and PI3K/AKT, respectively) might contribute an ‘enabling’ mechanism that augments Aβ toxicity. In this scenario, the protective function of small molecule ligands would be derived from optimizing the degenerative- versus survival-promoting functions of p75^NTR^ rather than inhibiting Aβ oligomer binding. The findings that NGF has no protective effect against Aβ in some assays, and that the toxic effect of Aβ is only partially lost in p75^NTR^−/− neurons, suggest that the protective effect of p75^NTR^ small molecule ligands, especially in assays in which toxic effects of Aβ are blocked entirely, is unlikely to be mediated solely by a simple mechanism of inhibiting Aβ binding to p75^NTR^.

Deleterious functional effects promoted by nanomolar, non-lethal, concentrations of Aβ may occur through mechanisms in addition to those promoting gross neuronal dystrophy and death. Recent findings suggest that p75^NTR^ is highly enriched in post-synaptic densities and interacts with the PDZ3 domain of the PSD-95 scaffolding protein [39]. In addition, evidence continues to accumulate that Aβ might impair synaptic function by perturbing PSD-95 related structures and function [40], [41]. Thus, the finding that LM11A-31 is capable of blocking low concentration Aβ-induced impairment of synaptic function in adult hippocampal slices suggests additional pathways through which p75^NTR^ small molecule ligands may mitigate Aβ effects. LM11A-31 is the first small molecule ligand shown to prevent Aβ-induced synaptic LTP impairment when administered at nanomolar, rather than micromolar, concentrations.

The favorable drug development profile of LM11A-24 and -31, their low nanomolar potency and receptor selectivity, and their unprecedented ability to simultaneously inhibit Aβ-induced activation of calpain/cdk5, GSK3β and c-Jun and to block Aβ impairment of synaptic function, each of which are current pharmaceutical candidate targets for AD therapeutics, establish an important new class of candidate small molecule compounds for AD therapeutics. Novel derivatives of these compounds with optimized pharmaceutical profiles are currently under study and will serve as important candidates for clinical development. Current studies in our laboratories are assessing the ability of p75^NTR^ ligands to decrease neurite degeneration and to correct behavioral deficits in transgenic mice that over-express Aβ.

## Methods

### Materials

All chemicals were purchased from Sigma (St Louis, MO), unless otherwise stated. The PI3-kinase inhibitor LY294002 was obtained from Calbiochem (La Jolla, CA). Anti-phospho-GSK3β^Ser9^ polyclonal antibody, anti-phospho-AKT monoclonal antibody, anti-phospho-CREB polyclonal antibody, anti-monoclonal CREB antibody, anti-AKT polyclonal antibody and anti-phospho-c-Jun polyclonal antibody were purchased from Cell Signaling (Beverly, MA). Monoclonal anti-GSK3β was obtained from BIOSOURCE International (Camarillo, CA). Monoclonal anti-phospho-tau^Ser202^ was purchased from Pierce (Rockford, IL). Polyclonal anti-tau, monoclonal microtubulin-associated protein-2 (MAP2) and monoclonal anti-actin antibodies were obtained from Sigma. α-fodrin and p35/p25 antibodies were procured from Santa Cruz Biotechnology (Santa, Cruz, CA). Mouse submaxillary NGF was obtained from Invitrogen (San Diego, CA).

### Primary neuronal cultures

Animal procedures were approved by each participating university's Committee on Laboratory Animal Care and were conducted in accordance with the NIH Guide for the care and use of laboratory animals. Cortical, hippocampal and septal cultures were prepared from embryonic day 16 (E16) CF1 mouse fetuses [42]. Tissue culture wells with or without coverslips were coated with 10 µg/mL poly-L-lysine in PBS. Cells were incubated in DMEM/F12 containing 10% fetal bovine serum for the first 16-20 hours, and subsequently maintained in serum-free Neurobasal medium with 1× B27 supplement (Invitrogen). p75^NTR^+/+ or −/− hippocampal neurons were derived from E15-17 mice maintained on a C57Bl/6 background and cultured under the same conditions, with the addition of 1 mM Glutamax supplement. For neuronal viability assays, neurons were seeded in 24-well plates at a density of 20,000–30,000 cells per well or in 12-well plates at 80,000–100,000 cells per well and allowed to mature 6–7 days. For neuritic dystrophy assays, 150,000–200,000 neurons per well were seeded into 6-well plates containing 25 mm coverslips and matured 21–22 days. For all cultures, medium was changed every 48–72 hours, prior to the addition of Aβ and various compounds. MAP2 immunostaining demonstrated that each of the above protocols resulted in cultures containing 90–95% neurons with 100% of the MAP2-positive cells expressing p75^NTR^. For Western blot signaling assays, the same protocol was followed except neurons were seeded at 450,000 cells per well in 6-well plates.

### Aβ preparations

Aβ(1-42), referred to as “Aβ”, and Aβ(S) (Aβ(1-42) residues in a scrambled sequence) were obtained from rPeptide (Athens, GA). Aβ oligomers were prepared using three different established methods. For the first two, Aβ peptide was resuspended in 0.5 mM NaOH [43] or 0.2% NH_4_OH [44] at a concentration of 350 µM and stored at −70°C. For use in cell cultures, the stock solution was incubated at 37°C for 5–7 days. In the third method [45], 1.0 mg of Aβ peptide was dissolved in 250 µl hexafluoroisopropanol (HFIP), aliquoted in sterile microcentrifuge tubes and HFIP was removed under vacuum in a Speed Vac. Resulting peptide films were stored desiccated at −20°C. Before use, the peptide was resuspended to 5 mM in dry dimethyl sulfoxide (Me_2_SO, Sigma), brought to 80 µM in PBS and incubated at 4°C for 16–24 hours. For the production of Aβ fibrils, HFIP-prepared peptide was brought to 222 µM in 10 mM HCl.

Peptide preparations were characterized by Atomic Force Microscopy (AFM). A multimode scanning probe microscope controlled by a NanoScope IIIa controller was used in conjunction with an E-series piezoelectric scanner (Digital Instruments, Santa Barbara, CA). AFM probes were etched silicon micro cantilevers, model MPP-11100 (Veeco, Santa Barbara, CA). Samples were applied to freshly cleaved mica using a Langmuir-Schaffer horizontal transfer system, and were then rinsed with Milli-Q Ultrapure water (Millipore, Temecula, CA) [46]. Image data was acquired at a scan rate of 0.5–1 Hz. AFM analysis (**Fig. S2**) of Aβ preparations demonstrated that NaOH- and NH_4_OH-based protocols resulted in primarily oligomeric species with occasional fibrils and that HFIP-based protocols resulted in primarily oligomeric species with rare or absent fibrils. The HCl-based protocol yielded primarily fibrillar species. Oligomeric Aβ preparations were used in all studies except as indicated in **Fig. 1J**.

### Quantitation of neuronal survival

p75^NTR^ ligands (LM11A-24 and LM11A-31), NGF or signaling inhibitors were added concomitantly with Aβ. Aβ preparations were added to 6–7 DIV cultures at a final concentration of 5 or 10 µM followed by 72 hours incubation. Following incubation with Aβ neurons were stained with Syto 13 (Molecular Probes), Hoechst 33258 (Calbiochem); or TUNEL/DAPI, using the fluorescein-12-dUTP, DeadEnd™ Fluorometric TUNEL System (Promega Madison, WI), and VECTASHIELD®+DAPI (Vector Labs Burlingame, CA). Stained neurons were visualized under a fluorescence microscope (Leica DM IRE2) using 520 nm (TUNEL, Syto13) or 460 nm (DAPI, Hoechst) filters. Survival of neurons was determined based on morphological criteria and Syto-13 (aids in cellular visualization) as assessed by phase contrast microscopy [47]. A dead or degenerating neuron was defined as one with a vacuolated cytoplasm, shrunken soma and/or beaded or retracted neurites. Data are expressed as percent of the total number of observed neurons that were scored as surviving. Neuron death was quantified with Hoechst 33258 by counting the number of cells containing condensed or fragmented nuclei as a percent of total observed neurons, and also with the TUNEL/DAPI system, by dividing the number of nuclei exhibiting TUNEL staining by the total number of nuclei as identified by DAPI. Cell survival analyses were confirmed by blinded counts.

### Quantitation of neuritic dystrophy

21–22 DIV hippocampal neuron cultures were treated with fresh Neurobasal B27 medium containing Aβ at a final concentration of 5 µM in the presence or absence of LM11A-24 or -31 for 48 hours and then fixed in fresh 4% paraformaldehyde. Neurites were imaged by immunostaining with MAP-2 monoclonal primary followed by Cy3-conjugated anti-mouse secondary antibody (Jackson Immunoresearch). MAP2 positive dendrites were quantitated using the MEASURE COUNT OBJECT function of MetaMorph 5.0 (Universal Imaging Inc, West Chester, PA). Only dendrite branches longer than 10 µm were considered (processes shorter than 10 µm were considered as sprouts). Dendrites were considered dystrophic when they showed a persistent pattern of increased tortuosity (multiple abrupt turns). To more precisely quantitate the degree of neurite curvature, we modified an established method for assessment of neurite curvature [31]. In randomly selected fields, neurite courses were digitized and approximated by a series of *n* manually chosen connected line segments using NIH-Image (**Fig. S4**). Neurite analyses were confirmed in a blinded manner. Using a Sigmaplot macro, the angle of each segment was determined relative to a line connecting the endpoints of the neurite tracing (a), and, beginning at one end, successive angles were subtracted from the prior angle in the chain, and the results averaged to give the ‘mean differential curvature’ (MDC = Σ(a_i+1_−a_i_)/n). This parameter reflects the degree of curvature over the course of the neurite with an increasing value indicating increased curvature.

### Organotypic hippocampal slice cultures

Organotypic hippocampal slice cultures were prepared using previously described methods [48] with modification. Briefly, 350 µm thick hippocampal slices were prepared from postnatal 8-day-old (PND-8) *Wistar* rats using a tissue chopper and separated in ice-cold Hank's balanced salt solution (HBSS) with 33.3 mM glucose, 4.2 mM NaHPO_4_, 10 mM MgSO_4_, 10 mM HEPES, 0.3% BSA and Penicillin-Streptomycin, pH 7.3. Slices were mounted on Millicell culture inserts and transferred to 6-well culture plates. Each well contained 1 mL of tissue culture medium consisting of 50% minimum essential medium, 25% HBSS, 25% heat inactivated horse serum and supplemented with 33 mM glucose, 12.5 mM HEPES and penicillin-streptomycin. Prior to the addition of Aβ and small molecules, organotypic cultures were matured under tissue culture conditions for 11–19 days. Culture medium was changed three times per week.

Pyramidal neuron death in brain slices was quantitated by measuring propidium iodide (PI) uptake using a previously established protocol [48]. In brief, slices were treated with Aβ±LM11A-31 for 24 hours and then with 50 µM N-Methyl-D-Aspartic acid (NMDA) to induce maximum pyramidal neuron death in each slice. Cellular damage was assessed by fluorescent image analysis of propidium iodide uptake (PI; 2 µg/mL), indicative of significant membrane injury. Before and after treatment, slices were observed and photographed with an inverted microscope (Nikon) coupled to a CCD camera, and mean fluorescent intensity in the CA1-3 regions was determined using OpenLab 4.04 software. Baseline (i.e. pre-treatment) fluorescence was subtracted from subsequent measurements and post-treatment cellular damage was normalized to the total neuronal density (determined by NMDA-induced PI intensity).

### Protein preparation and Western blotting

For assays of AKT, calpain, cdk5 and GSK3β activity, 21–22 DIV hippocampal neurons were incubated for 4 hours in fresh neurobasal/B27 culture medium containing 5 µM oligomeric Aβ in the presence or absence of LM11A-24, -31 or NGF. Following incubation, cells were collected and washed in ice-cold PBS and then lysed in RIPA buffer (20 mM Tris, pH 8.0, 137 mM NaCl, 1% NP-40, 10% glycerol, 1 mM PMSF, 500 µM orthovanadate, 10 µg/ml aprotinin and 1 µg/ml leupeptin). Lysates were sonicated for 10 seconds, centrifuged at 14,000×*g* for 10 minutes at 4°C, and the supernatant was collected. For tau phosphorylation studies, heat-stable fractions were prepared as described previously [49]. In brief, cells were collected by scraping cells into ice-cold TBS and centrifuging at 14000×*g* for 10 min at 4°C. The supernatant was discarded and the pellet was resuspended in 100 µl MES/NaCl buffer (100 mM MES, 1 M NaCl, 0.5 mM MgCl_2_, 1 mM EGTA, 2 mM DTT, 1 mM Na_3_VO_4_, 1 mM benzamidine hydrochloride, 5 µg/ml leupeptin, 2 µg/ml aprotinin, 1 µg/ml pepstatin, 0.2 mM PMSF) and immediately heated to 100°C for 10 min. These were then cooled on ice and centrifuged at 14000×*g* at 4°C for 25 min. The tau and MAP2c-enriched supernatant was retained. Phosphorylated CREB (p-CREB) levels were determined as described previously [21]. Cultured 21–22 DIV hippocampal neurons were treated with 5 µM Aβ in Neurobasal medium without B27 supplement or L-glutamine for 3 hours followed by the addition of 50 µM glutamine for 15 min in order to promote CREB activation (phosphorylation). Cells were harvested in 1× modified RIPA buffer as above with the addition of 80 mM glycerophosphate, and whole-cell extracts were then prepared. Protein concentrations were determined using the BCA Protein Assay Reagent (Pierce, Rockford, IL). Samples were electrophoresed through 4–20% Tris-HCl Linear Gradient Gels (Bio-Rad, Hercules, CA) and transferred to PVDF membranes. Western blots were processed using the ECL Chemiluminescence System (Amersham, Arlington Heights, IL) and bands were quantitated using densitometry.

### Quantitation of c-Jun activation

E16 hippocampal neurons harvested from CF-1 mice were plated on glass coverslips as described above or on Tissue Tek chamber slides and at 6–7 DIV were treated with 5–10 µM oligomeric Aβ for 12 hours, then fixed in fresh 4% PFA and immunostained with phospho-c-Jun specific antibody and DAPI or Hoechst to label total nuclei [35]. Activation of c-Jun was quantitated by counting p-c-Jun positive nuclei as a percent of total neuronal nuclei.

### Electrophysiological recordings

Hippocampal slice preparations were performed as described [21]. Hippocampi were harvested from 3 month old male mice (C57Bl/6; The Jackson Laboratory). Transverse hippocampal slices (400 µm in thickness) were cut and maintained in an interface chamber at 29°C, and perfused with saline solution (124.0 mM NaCL, 4.4 mM KCL, 1.0 mM Na_2_HPO_4_, 25.0 mM NaHCO_3_, 2.0 mM CaCL_2_, 2.0 mM MgSO_4_, and 10 mM glucose) continuously bubbled with 95% O_2_ and 5% CO_2_. fEPSPs were recorded from the CA1 region of the hippocampus by placement of both the stimulating and the recording electrodes in the CA1 stratum radiatum. Basal synaptic transmission (BST) was assayed by plotting of the stimulus voltage (V) against slopes of fEPSP to generate input-output relations or by plotting of the peak amplitude of the fiber volley against the slope of the fEPSP to generate input-output relations. LTP was induced using theta-burst stimulation (4 pulses at 100 Hz, with the bursts repeated at 5 Hz, and each tetanus including 3 10-burst trains separated by 15 seconds). LM11A-31 (100 nM) (dissolved in H_2_O) or vehicle (H_2_O) was added to the bath solution for 20 minutes prior to the induction of LTP in studies in which Aβ (200 nM) was added to slice preparations. Oligomeric Aβ(1-42) was prepared using HFIP [45].

### Statistical analysis

Statistical analyses applied ANOVA with Dunnett's correction except for the following: in **Fig. 2** and **Fig. 5B** ANOVA with Tukey-Kramer correction was applied; and in **Fig. 6** two-way ANOVA was applied. For all bar graphs, mean±SE is shown. For all figures: *p<0.05, **p<0.01, ***p<0.001.

## Supporting Information

Figure S1 Small molecule structures. Structures of LM11A-31, LM11A-24 and LM11A-36 are shown. LM11A-36 is identical to LM11A-24 except that it contains two additional methyl groups and is inactive. These compound structures were published previously [17].
(0.10 MB TIF)

Figure S2 AFM imaging of Aβ preparations. Images are representative ∼1×1 µm scans with z-height 10 nm. Incubation Aβ(1-42) peptide in NaOH (A) or NH4OH (B) demonstrates primarily oligomeric structures with occasional fibrillar structures. (C) Aβ preparation derived from HFIP-processing of Aβ followed by PBS incubation demonstrates oligomeric structure with no observed fibrils. (D) Aβ fibril preparation derived from HFIP-processing of Aβ followed by HCl incubation demonstrates primarily fibril-like structures.
(6.83 MB TIF)

Figure S3 LM11A-24 and -31 inhibit Aβ-induced death of hippocampal neurons as assayed by Hoechst staining. 6–7 DIV hippocampal neurons were treated with (A) culture medium (CM) alone; (B) 10 µM Aβ; (C) 10 µM Aβ with 100 nM LM11A-24; (D) 10 µM Aβ with 100 nM LM11A-31 for 72 hours, then stained with Hoechst 33258, fixed and photographed with fluorescence microscopy. Many neurons exposed to Aβ exhibited nuclear condensation and fragmentation, indicative of death (arrows in B), whereas the majority of neurons co-treated with Aβ and LM11A-24 or -31 had diffuse, even nuclei, similar to the control condition. (E) Treatment with Aβ resulted in an approximately 4-fold increase in neuronal death. Co-treatment with 100 nM LM11A-24 or -31, but not NGF, prevented Aβ-induced death (n = 18–58 fields derived from 3–6 separate experiments). Each condition was compared to Aβ alone.
(0.65 MB TIF)

Figure S4 Illustration of neurite curvature quantitation. As described in Methods, neurites were traced manually in the form of a series of short vectors (small arrows along neurites) to determine the angles (ai, ai+1…) created by each vector and a line connecting the origin and termination of the measured segment. The differences between successive angles (e.g. ai+1-ai) were averaged to generate a mean differential curvature (MDC) score. The upper panel demonstrates a neurite in culture medium alone (CM) with a mean differential curvature of 10.7 and the lower panel demonstrates a neurite exposed to Aβ with a mean differential curvature of 29.3.
(1.92 MB TIF)

Table S1 Cerep receptor screen. Compound LM11A-31 was submitted to Cerep Inc. (Seattle, WA) and applied to the ExpresSProfile receptor screen. Values shown indicate the percent by which binding of a control ligand is inhibited by the test compound. Inhibition of binding by <20% is interpreted as no significant binding detected.
(0.10 MB DOC)

## References

[1] Coulson EJ (2006). Does the p75 neurotrophin receptor mediate Abeta-induced toxicity in Alzheimer's disease?. J Neurochem.

[2] Longo FM, Yang T, Knowles JK, Xie Y, Moore LA (2007). Small molecule neurotrophin receptor ligands: novel strategies for targeting Alzheimer's disease mechanisms.. Curr Alzheimer Res.

[3] Roux PP, Barker PA (2002). Neurotrophin signaling through the p75 neurotrophin receptor.. Prog Neurobiol.

[4] Dechant G, Barde YA (2002). The neurotrophin receptor p75(NTR): novel functions and implications for diseases of the nervous system.. Nat Neurosci.

[5] Longo FM, Massa SM (2008). Small Molecule Modulation of p75 Neurotrophin Receptor Functions.. CNS Neurol Disord Drug Targets.

[6] Mufson EJ, Kordower JH (1992). Cortical neurons express nerve growth factor receptors in advanced age and Alzheimer disease.. Proc Natl Acad Sci U S A.

[7] Hu XY, Zhang HY, Qin S, Xu H, Swaab DF (2002). Increased p75(NTR) expression in hippocampal neurons containing hyperphosphorylated tau in Alzheimer patients.. Exp Neurol.

[8] Cruz JC, Tsai LH (2004). Cdk5 deregulation in the pathogenesis of Alzheimer's disease.. Trends Mol Med.

[9] Balaraman Y, Limaye AR, Levey AI, Srinivasan S (2006). Glycogen synthase kinase 3beta and Alzheimer's disease: pathophysiological and therapeutic significance.. Cell Mol Life Sci.

[10] Thakur A, Wang X, Siedlak SL, Perry G, Smith MA (2007). c-Jun phosphorylation in Alzheimer disease.. J Neurosci Res.

[11] Coulson EJ, Reid K, Baca M, Shipham KA, Hulett SM (2000). Chopper, a new death domain of the p75 neurotrophin receptor that mediates rapid neuronal cell death.. J Biol Chem.

[12] Arevalo MA, Rodriguez-Tebar A (2006). Activation of casein kinase II and inhibition of phosphatase and tensin homologue deleted on chromosome 10 phosphatase by nerve growth factor/p75NTR inhibit glycogen synthase kinase-3beta and stimulate axonal growth.. Mol Biol Cell.

[13] Linggi MS, Burke TL, Williams BB, Harrington A, Kraemer R (2005). Neurotrophin receptor interacting factor (NRIF) is an essential mediator of apoptotic signaling by the p75 neurotrophin receptor.. J Biol Chem.

[14] Yaar M, Zhai S, Panova I, Fine RE, Eisenhauer PB (2007). A cyclic peptide that binds p75(NTR) protects neurones from beta amyloid (1-40)-induced cell death.. Neuropathol Appl Neurobiol.

[15] Rabizadeh S, Bitler CM, Butcher LL, Bredesen DE (1994). Expression of the low-affinity nerve growth factor receptor enhances beta-amyloid peptide toxicity.. Proc Natl Acad Sci U S A.

[16] Townsend M, Mehta T, Selkoe DJ (2007). Soluble Abeta inhibits specific signal transduction cascades common to the insulin receptor pathway.. J Biol Chem.

[17] Massa SM, Xie Y, Yang T, Harrington AW, Kim ML (2006). Small, nonpeptide p75NTR ligands induce survival signaling and inhibit proNGF-induced death.. J Neurosci.

[18] Wei W, Wang X, Kusiak JW (2002). Signaling events in amyloid beta-peptide-induced neuronal death and insulin-like growth factor I protection.. J Biol Chem.

[19] Cross DA, Alessi DR, Cohen P, Andjelkovich M, Hemmings BA (1995). Inhibition of glycogen synthase kinase-3 by insulin mediated by protein kinase B.. Nature.

[20] Mazanetz MP, Fischer PM (2007). Untangling tau hyperphosphorylation in drug design for neurodegenerative diseases.. Nat Rev Drug Discov.

[21] Vitolo OV, Sant'Angelo A, Costanzo V, Battaglia F, Arancio O (2002). Amyloid beta -peptide inhibition of the PKA/CREB pathway and long-term potentiation: reversibility by drugs that enhance cAMP signaling.. Proc Natl Acad Sci U S A.

[22] Wang Q, Walsh DM, Rowan MJ, Selkoe DJ, Anwyl R (2004). Block of long-term potentiation by naturally secreted and synthetic amyloid beta-peptide in hippocampal slices is mediated via activation of the kinases c-Jun N-terminal kinase, cyclin-dependent kinase 5, and p38 mitogen-activated protein kinase as well as metabotropic glutamate receptor type 5.. J Neurosci.

[23] Zhu LQ, Wang SH, Liu D, Yin YY, Tian Q (2007). Activation of glycogen synthase kinase-3 inhibits long-term potentiation with synapse-associated impairments.. J Neurosci.

[24] Borsello T, Forloni G (2007). JNK signalling: a possible target to prevent neurodegeneration.. Curr Pharm Des.

[25] Yoshida H, Hastie CJ, McLauchlan H, Cohen P, Goedert M (2004). Phosphorylation of microtubule-associated protein tau by isoforms of c-Jun N-terminal kinase (JNK).. J Neurochem.

[26] Longo FM, Manthorpe M, Xie YM, Varon S (1997). Synthetic NGF peptide derivatives prevent neuronal death via a p75 receptor-dependent mechanism.. J Neurosci Res.

[27] Friedman WJ (2000). Neurotrophins induce death of hippocampal neurons via the p75 receptor.. J Neurosci.

[28] Nassif M, Hoppe J, Santin K, Frozza R, Zamin LL (2007). Beta-amyloid peptide toxicity in organotypic hippocampal slice culture involves Akt/PKB, GSK-3beta, and PTEN.. Neurochem Int.

[29] Heredia L, Helguera P, de Olmos S, Kedikian G, Sola Vigo F (2006). Phosphorylation of actin-depolymerizing factor/cofilin by LIM-kinase mediates amyloid beta-induced degeneration: a potential mechanism of neuronal dystrophy in Alzheimer's disease.. J Neurosci.

[30] Ferreira A, Lu Q, Orecchio L, Kosik KS (1997). Selective phosphorylation of adult tau isoforms in mature hippocampal neurons exposed to fibrillar A beta.. Mol Cell Neurosci.

[31] Knowles RB, Wyart C, Buldyrev SV, Cruz L, Urbanc B (1999). Plaque-induced neurite abnormalities: implications for disruption of neural networks in Alzheimer's disease.. Proc Natl Acad Sci U S A.

[32] Lee YB, Du S, Rhim H, Lee EB, Markelonis GJ (2000). Rapid increase in immunoreactivity to GFAP in astrocytes in vitro induced by acidic pH is mediated by calcium influx and calpain I.. Brain Res.

[33] Wang KK (2000). Calpain and caspase: can you tell the difference?. Trends Neurosci.

[34] Levresse V, Butterfield L, Zentrich E, Heasley LE (2000). Akt negatively regulates the cJun N-terminal kinase pathway in PC12 cells.. J Neurosci Res.

[35] Harris CA, Deshmukh M, Tsui-Pierchala B, Maroney AC, Johnson EM (2002). Inhibition of the c-Jun N-terminal kinase signaling pathway by the mixed lineage kinase inhibitor CEP-1347 (KT7515) preserves metabolism and growth of trophic factor-deprived neurons.. J Neurosci.

[36] Hashiguchi M, Saito T, Hisanaga S, Hashiguchi T (2002). Truncation of CDK5 activator p35 induces intensive phosphorylation of Ser202/Thr205 of human tau.. J Biol Chem.

[37] Li T, Paudel HK (2006). Glycogen synthase kinase 3beta phosphorylates Alzheimer's disease-specific Ser396 of microtubule-associated protein tau by a sequential mechanism.. Biochemistry.

[38] Cragnolini AB, Friedman WJ (2008). The function of p75NTR in glia.. Trends Neurosci.

[39] Sandoval M, Sandoval R, Thomas U, Spilker C, Smalla KH (2007). Antagonistic effects of TrkB and p75(NTR) on NMDA receptor currents in post-synaptic densities transplanted into Xenopus oocytes.. J Neurochem.

[40] Shankar GM, Bloodgood BL, Townsend M, Walsh DM, Selkoe DJ (2007). Natural oligomers of the Alzheimer amyloid-beta protein induce reversible synapse loss by modulating an NMDA-type glutamate receptor-dependent signaling pathway.. J Neurosci.

[41] Lacor PN, Buniel MC, Furlow PW, Clemente AS, Velasco PT (2007). Abeta oligomer-induced aberrations in synapse composition, shape, and density provide a molecular basis for loss of connectivity in Alzheimer's disease.. J Neurosci.

[42] Yang T, Bernabeu R, Xie Y, Zhang JS, Massa SM (2003). Leukocyte antigen-related protein tyrosine phosphatase receptor: a small ectodomain isoform functions as a homophilic ligand and promotes neurite outgrowth.. J Neurosci.

[43] Takahashi RH, Almeida CG, Kearney PF, Yu F, Lin MT (2004). Oligomerization of Alzheimer's beta-amyloid within processes and synapses of cultured neurons and brain.. J Neurosci.

[44] Bozyczko-Coyne D, O'Kane TM, Wu ZL, Dobrzanski P, Murthy S (2001). CEP-1347/KT-7515, an inhibitor of SAPK/JNK pathway activation, promotes survival and blocks multiple events associated with Abeta-induced cortical neuron apoptosis.. J Neurochem.

[45] Stine WB, Dahlgren KN, Krafft GA, LaDu MJ (2003). In vitro characterization of conditions for amyloid-beta peptide oligomerization and fibrillogenesis.. J Biol Chem.

[46] Widenbrant MJ, Rajadas J, Sutardja C, Fuller GG (2006). Lipid-induced beta-amyloid peptide assemblage fragmentation.. Biophys J.

[47] Eichler ME, Dubinsky JM, Rich KM (1992). Relationship of intracellular calcium to dependence on nerve growth factor in dorsal root ganglion neurons in cell culture.. J Neurochem.

[48] McCullough L, Wu L, Haughey N, Liang X, Hand T (2004). Neuroprotective function of the PGE2 EP2 receptor in cerebral ischemia.. J Neurosci.

[49] Williamson R, Scales T, Clark BR, Gibb G, Reynolds CH (2002). Rapid tyrosine phosphorylation of neuronal proteins including tau and focal adhesion kinase in response to amyloid-beta peptide exposure: involvement of Src family protein kinases.. J Neurosci.

