# Supplementary figures and images for: Small Molecule, Non-Peptide p75NTR Ligands Inhibit Aβ-Induced Neurodegeneration and Synaptic Impairment
Source: PLoS One. 2008 Nov 3;3(11):e3604. doi: 10.1371/journal.pone.0003604 (PMC2575383; doi:10.1371/journal.pone.0003604)

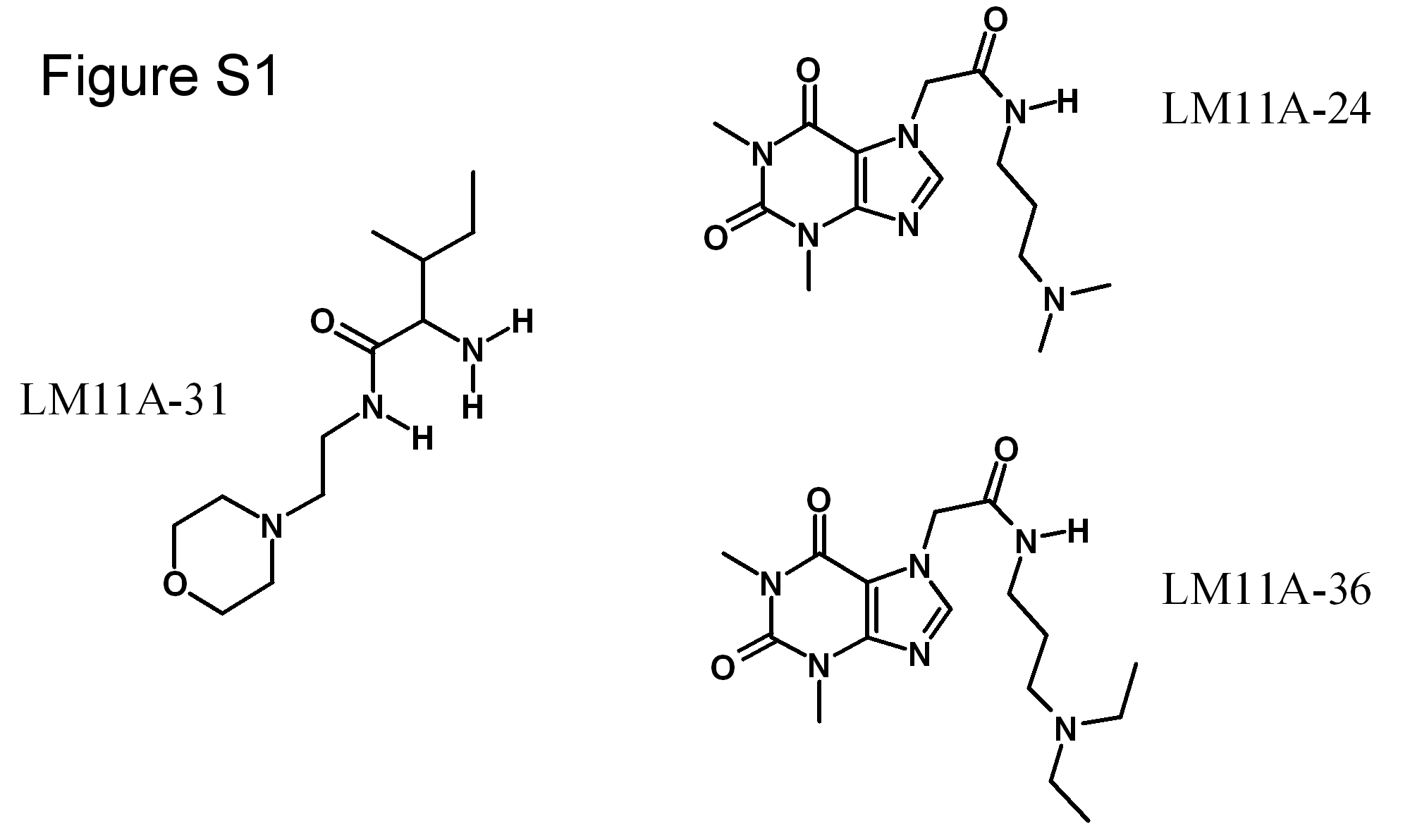

Supplement: Figure S1 — Small molecule structures. Structures of LM11A-31, LM11A-24 and LM11A-36 are shown. LM11A-36 is identical to LM11A-24 except that it contains two additional methyl groups and is inactive. These compound structures were published previously [17]. (0.10 MB TIF) [file pone.0003604.s001.tif]

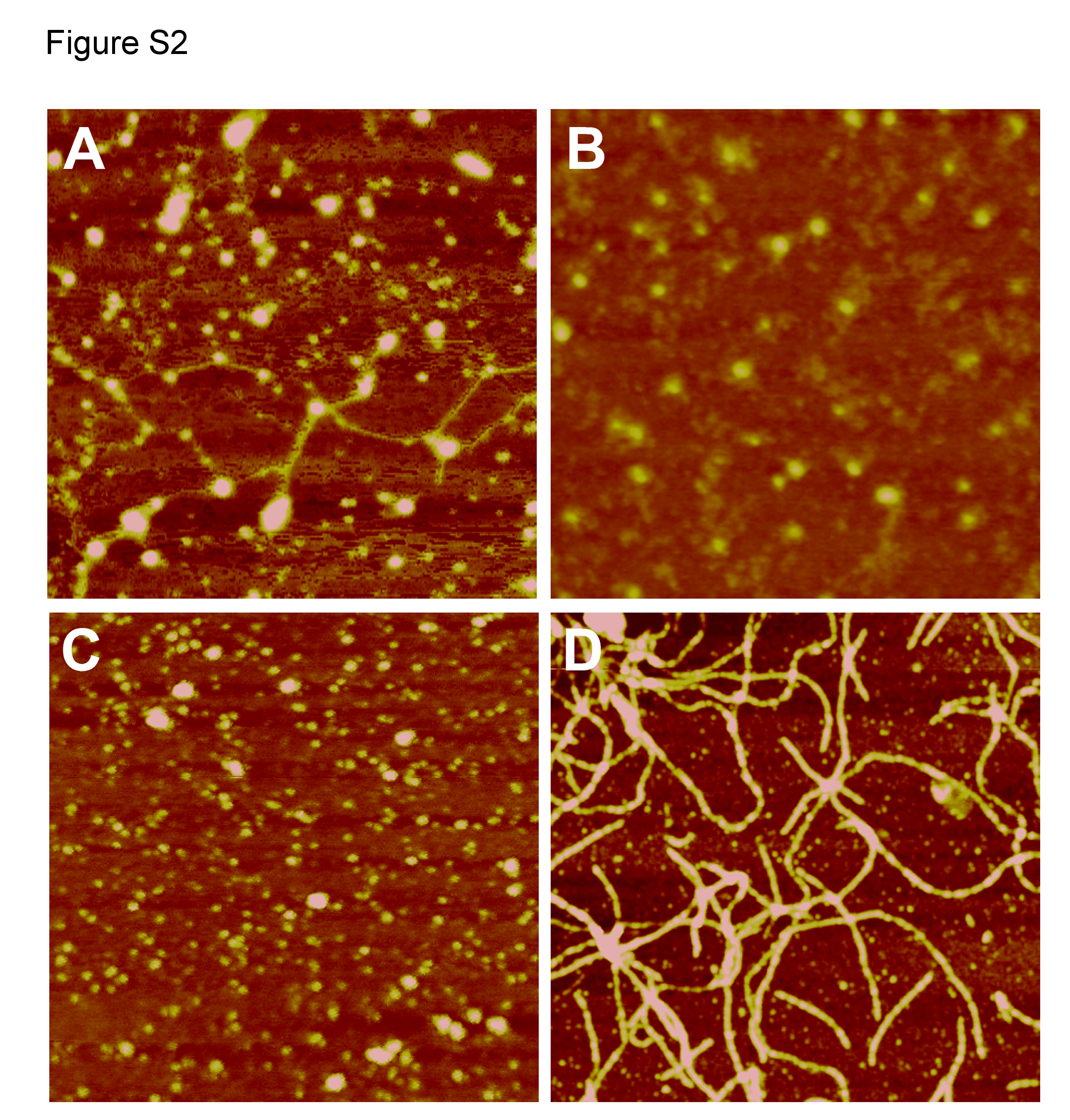

Supplement: Figure S2 — AFM imaging of Aβ preparations. Images are representative ∼1×1 µm scans with z-height 10 nm. Incubation Aβ(1-42) peptide in NaOH (A) or NH4OH (B) demonstrates primarily oligomeric structures with occasional fibrillar structures. (C) Aβ preparation derived from HFIP-processing of Aβ followed by PBS incubation demonstrates oligomeric structure with no observed fibrils. (D) Aβ fibril preparation derived from HFIP-processing of Aβ followed by HCl incubation demonstrates primarily fibril-like structures. (6.83 MB TIF) [file pone.0003604.s002.tif]

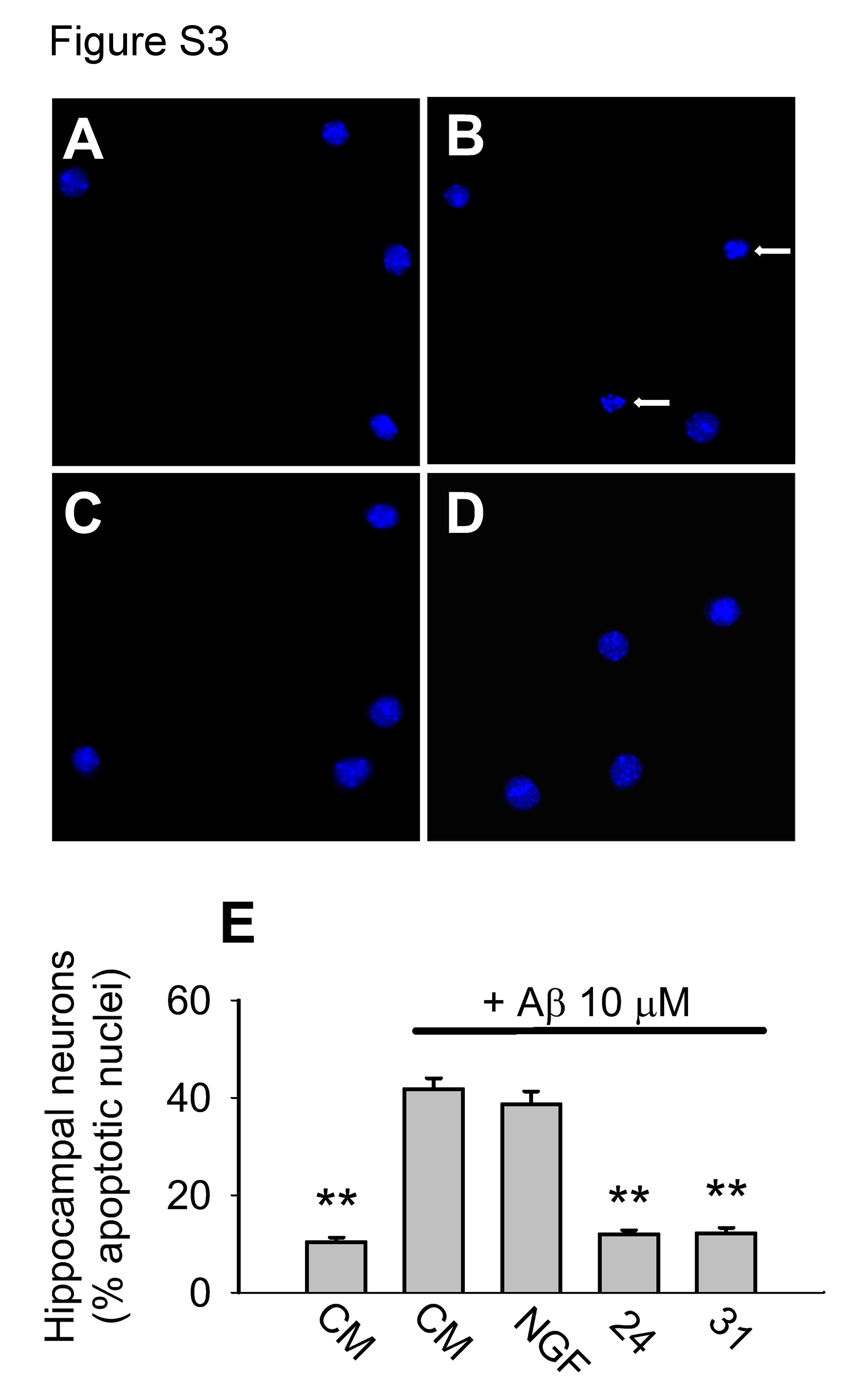

Supplement: Figure S3 — LM11A-24 and -31 inhibit Aβ-induced death of hippocampal neurons as assayed by Hoechst staining. 6–7 DIV hippocampal neurons were treated with (A) culture medium (CM) alone; (B) 10 µM Aβ; (C) 10 µM Aβ with 100 nM LM11A-24; (D) 10 µM Aβ with 100 nM LM11A-31 for 72 hours, then stained with Hoechst 33258, fixed and photographed with fluorescence microscopy. Many neurons exposed to Aβ exhibited nuclear condensation and fragmentation, indicative of death (arrows in B), whereas the majority of neurons co-treated with Aβ and LM11A-24 or -31 had diffuse, even nuclei, similar to the control condition. (E) Treatment with Aβ resulted in an approximately 4-fold increase in neuronal death. Co-treatment with 100 nM LM11A-24 or -31, but not NGF, prevented Aβ-induced death (n = 18–58 fields derived from 3–6 separate experiments). Each condition was compared to Aβ alone. (0.65 MB TIF) [file pone.0003604.s003.tif]

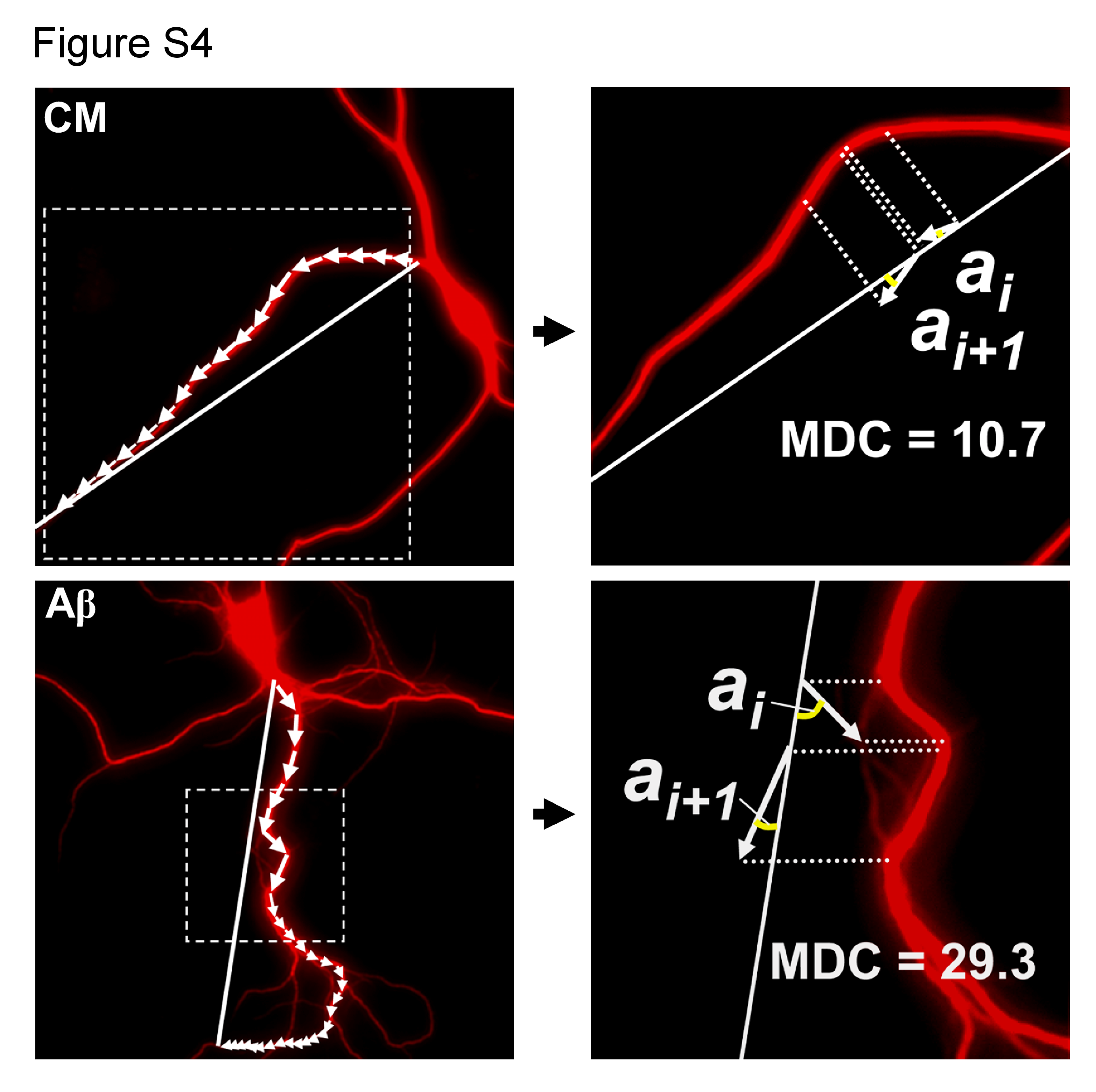

Supplement: Figure S4 — Illustration of neurite curvature quantitation. As described in Methods, neurites were traced manually in the form of a series of short vectors (small arrows along neurites) to determine the angles (ai, ai+1…) created by each vector and a line connecting the origin and termination of the measured segment. The differences between successive angles (e.g. ai+1-ai) were averaged to generate a mean differential curvature (MDC) score. The upper panel demonstrates a neurite in culture medium alone (CM) with a mean differential curvature of 10.7 and the lower panel demonstrates a neurite exposed to Aβ with a mean differential curvature of 29.3. (1.92 MB TIF) [file pone.0003604.s004.tif]
