# Supplementary material for: Small Molecule, Non-Peptide p75NTR Ligands Inhibit Aβ-Induced Neurodegeneration and Synaptic Impairment
Source: PLoS One. 2008 Nov 3;3(11):e3604. doi: 10.1371/journal.pone.0003604 (PMC2575383; doi:10.1371/journal.pone.0003604)
Supplement: Table S1 — Cerep receptor screen. Compound LM11A-31 was submitted to Cerep Inc. (Seattle, WA) and applied to the ExpresSProfile receptor screen. Values shown indicate the percent by which binding of a control ligand is inhibited by the test compound. Inhibition of binding by <20% is interpreted as no significant binding detected. (0.10 MB DOC) [file pone.0003604.s005.doc]

Table S1. Cerep receptor screen.

| Cerep Compound I.D. | % Inhibition of Control Specific Binding |
| --- | --- |
|  |  |
| A1 (h) | -10 |
|  |  |
| A2A (h) | -9 |
|  |  |
| A3 (h) | -6 |
|  |  |
| α1 (non-selective) | 4 |
|  |  |
| α2 (non-selective) | -4 |
|  |  |
| β1 (h) | -1 |
|  |  |
| β2 (h) | -3 |
|  |  |
| AT1 (h) | -7 |
|  |  |
| BZD (central) | -2 |
|  |  |
| B2 (h) | -5 |
|  |  |
| CB1 (h) | 11 |
|  |  |
| CCKA (h) (CCK1) | -17 |
|  |  |
| D1 (h) | -2 |
|  |  |
| D2S (h) | 8 |
|  |  |
| ETA (h) | 4 |
|  |  |
| GABA (non-selective) | -2 |
|  |  |
| GAL2 (h) | 3 |
|  |  |
| CXCR2 (h) (IL-8B) | -2 |
|  |  |
| CCR1 (h) | 0 |
|  |  |
| Ghrelin (h) (GHS) | -8 |
|  |  |
| H1 (h) | -2 |
|  |  |
| H2 (h) | 7 |
|  |  |
| MC4 (h) | -7 |
|  |  |
| MT1 (h) | 1 |
|  |  |
| M1 (h) | -2 |
|  |  |
| M2 (h) | -3 |
|  |  |
| M3 (h) | 0 |
|  |  |
| NK2 (h) | -6 |
|  |  |
| NK3 (h) | -2 |
|  |  |
| Y1 (h) | -13 |
|  |  |
| Y2 (h) | -3 |
|  |  |
| NT1 (h) (NTS1) | -3 |
|  |  |
| δ2 (h) (DOP) | -1 |
|  |  |
| κ (KOP) | 0 |
|  |  |
| μ (h) (MOP) (agonist site) | 10 |
|  |  |
| ORL1 (h) (NOP) | -8 |
|  |  |
| TXA2/PGH2 (h) (TP) | 1 |
|  |  |
| 5-HT1A (h) | -2 |
|  |  |
| 5-HT1B | -3 |
|  |  |
| 5-HT2A (h) | 1 |
|  |  |
| 5-HT3 (h) | 0 |
|  |  |
| 5-HT5A (h) | -2 |
|  |  |
| 5-HT6 (h) | -2 |
|  |  |
| 5-HT7 (h) | -3 |
|  |  |
| sst (non-selective) | -5 |
|  |  |
| VIP1 (h) (VPAC1) | 1 |
|  |  |
| V1a (h) | 2 |
|  |  |
| Ca2+ channel (L, verapamil site) (phenylalkylamines) | 5 |
|  |  |
| K+ V channel | -7 |
|  |  |
| SK+ Ca channel | -3 |
|  |  |
| Na+ channel (site 2) | 5 |
|  |  |
| Cl channel | -6 |
|  |  |
| NE transporter (h) | -9 |
|  |  |
| DA transporter (h) | 1 |
|  |  |
| 5-HT transporter (h) | 1 |
